# Friend and Confidant Thresholds: Social Network Size as a Mediator Between Marital Status and Major Depressive Disorder

**DOI:** 10.1155/da/5174978

**Published:** 2026-07-05

**Authors:** Han-Sung Lee, Yong Min Ahn, Sooyeon Min, Yoojin Song, Heon-Jeong Lee, Seunghee Won, Kyu Young Lee, Dongyun Lee, Do Hoon Kim, Ji Hyun Baek, Young-Hoon Ko, Kwang-Yeon Choi, Young Tak Jo, Sung Joon Cho, Hong Seok Oh, Won Sub Kang, Young Min Choe, Sungwon Roh, Hyun-Ju Kim, Joonho Choi, Ga Eun Kim, So-Hyun Ahn, Hyeon-Ah Lee, Kenneth Kendler, Sang Jin Rhee, Ji-Woon Jeong, Jonathan Flint

**Affiliations:** ^1^ Department of Neuropsychiatry, Seoul National University Hospital, Seoul, Republic of Korea, snuh.org; ^2^ Department of Psychiatry, Seoul National University College of Medicine, Seoul, Republic of Korea, snu.ac.kr; ^3^ Department of Psychiatry, Kangwon National University Hospital, Kangwon National University College of Medicine, Chuncheon, Republic of Korea, knuh.or.kr; ^4^ Department of Psychiatry, Korea University Anam Hospital, Seoul, Republic of Korea, kumc.or.kr; ^5^ Department of Psychiatry, School of Medicine, Kyungpook National University, Daegu, Republic of Korea, knu.ac.kr; ^6^ Department of Psychiatry, Nowon Eulji University Hospital, Seoul, Republic of Korea; ^7^ Department of Psychiatry, Gyeongsang National University Changwon Hospital, Gyeongsang National University School of Medicine, Jinju, Republic of Korea, gnu.ac.kr; ^8^ Department of Psychiatry, Chuncheon Sacred Heart Hospital and Mind-Neuromodulation Laboratory, Hallym University College of Medicine, Chuncheon, Republic of Korea, hallym.ac.kr; ^9^ Department of Psychiatry, Samsung Medical Center, Seoul, Republic of Korea, samsunghospital.com; ^10^ Department of Psychiatry, Korea University Ansan Hospital, Ansan, Republic of Korea, kumc.or.kr; ^11^ Department of Psychiatry, Chungnam National University College of Medicine, Chungnam National University Hospital, Daejeon, Republic of Korea, cnu.ac.kr; ^12^ Department of Psychiatry, Kangdong Sacred Heart Hospital, Seoul, Republic of Korea; ^13^ Department of Psychiatry, Kangbuk Samsung Hospital, Sungkyunkwan University School of Medicine, Seoul, Republic of Korea, skkumed.ac.kr; ^14^ Department of Psychiatry, Konyang University Hospital, Daejeon, Republic of Korea, konyang.ac.kr; ^15^ Department of Psychiatry, Kyung Hee University College of Medicine, Kyung Hee University Hospital, Seoul, Republic of Korea, khmc.or.kr; ^16^ Department of Neuropsychiatry, Hallym University Dongtan Sacred Heart Hospital, Hwaseong, Republic of Korea, hallym.or.kr; ^17^ Department of Psychiatry, Hanyang University Hospital, Seoul, Republic of Korea; ^18^ Department of Psychiatry, CHA Bundang Medical Center, CHA University School of Medicine, Seongnam, Republic of Korea, cha.ac.kr; ^19^ Department of Psychiatry, Hanyang University Guri Hospital, Guri, Republic of Korea, hanyang.ac.kr; ^20^ Department of Psychiatry, Ewha Womans University Mokdong Hospital, Ewha Womans University College of Medicine, Seoul, Republic of Korea, ewha.ac.kr; ^21^ Department of Psychiatry, Soonchunhyang University Cheonan Hospital, Cheonan, Republic of Korea, sch.ac.kr; ^22^ Department of Psychiatry, Virginia Commonwealth University, Richmond, Virginia, USA, vcu.edu; ^23^ Department of Psychiatry, Pusan National University Yangsan Hospital, Yangsan, Republic of Korea, pnuyh.or.kr; ^24^ Department of Psychiatry and Biobehavioral Sciences, University of California, Los Angeles, California, USA, berkeley.edu

**Keywords:** Korean Mood Disorder Genetic Study-Depression (KOMOGEN-D), major depressive disorder (MDD), marital status, social network, social support

## Abstract

**Background:**

Marriage rates are declining globally, a trend particularly notable in South Korea, where demographic shifts raise concerns about social well‐being. While marital status is consistently linked to mental health, the extent to which social network size mediates the association between marital status and depression, particularly among contemporary Korean women, remains insufficiently understood.

**Methods:**

We analyzed 5443 Korean women aged 40 or older from the Korean Mood Disorder Genetic Study‐Depression (KOMOGEN‐D) study (2394 recurrent major depressive disorder [MDD]; 3049 controls). Marital status was dichotomized into married and unmarried (never married, divorced/separated, widowed). Social network size was operationalized as the count of friends and confidants. We used multivariable logistic regression and parallel mediation analysis to examine associations. Exploratory piecewise logistic regression identified network size thresholds for MDD risk reduction.

**Results:**

Unmarried women showed nearly four‐fold higher odds of MDD (odds ratio [OR] 3.99), with the highest risk among divorced/separated women (OR 4.90). Increased social network size was associated with reduced MDD odds (per additional friend: OR 0.90; per additional confidant: OR 0.84, in a mutually adjusted model), and network size accounted for ~13% of this association. A protective baseline of approximately two friends and one confidant was observed in the overall sample; however, this threshold shifted upward for unmarried women (3–4 friends/confidants).

**Conclusions:**

Living without a spouse is strongly associated with MDD, partially due to smaller social networks. Maintaining a minimum core of supportive relationships is essential, suggesting that interventions to strengthen social networks are vital public health strategies for unmarried Korean women.

## 1. Introduction

Globally, marriage rates have declined substantially over the past few decades, dropping from 7 to 9 per 1000 people per year in the 1960 s to fewer than 5 per 1000 per year by 2020. The United States, for example, recorded a record‐low marriage rate of about 5.1 marriages per 1000 people in 2020 [1]. In South Korea, this shift is particularly notable: over the past four decades, the average age at first marriage has been delayed by more than 4 years for both men and women, while the crude marriage rate has continued to decrease [2–4]. Correspondingly, South Korea’s total fertility rate has remained below 1.3 since 2001, ranking among the lowest in the world [5]. These demographic changes have accelerated since the COVID‐19 pandemic [6]. As marriage becomes less common and more delayed, concerns are growing about how these shifts might reshape social structures and interpersonal well‐being. In this context, it is important to examine how remaining unmarried may affect individuals’ social lives and mental health.

The unmarried status has long been associated with poorer mental health. Recent international evidence indicates that unmarried people have higher levels of depressive symptoms than married people, and a meta‐analysis of adults aged 55 years or older similarly found a higher risk of depression among unmarried adults [7, 8]. Korean data show a comparable pattern, with women who remained unmarried beyond the mean marriage age reporting higher rates of mood disorders and suicidality than age‐matched married women [9]. However, neither the social meaning of marriage nor its association with health is fixed. Marriage has been progressively deinstitutionalized [10], and the strength of the association between marital status and health has itself varied across historical periods [11]. How strongly marital status relates to depression among women in contemporary Korea, and through what social pathways, therefore warrants direct examination.

One widely cited explanation for the protective effect of marriage is stronger social relationships and support. Marriage often provides a primary confidant, integrates the individual into a larger kin network, and can enhance both perceived and received social support. Cross‐sectional data from Singapore, for example, show that married adults report higher perceived support, which in turn is associated with lower odds of common mental disorders [12]. Conversely, a prospective study indicates that individuals with low social support or sustained social isolation face a markedly higher risk of developing major depressive disorder (MDD) [13]. Sex differences add nuance to this pathway. Women typically maintain broader and more intimate social networks than men yet appear more sensitive to the loss or absence of support [14, 15]. A twin cohort study showed that low global social support predicted future major depression significantly more strongly in women than in men [16].

These accounts, however, primarily concern the functional quality of support. By contrast, the structural size of a person’s network, namely, the number of ties available to draw on, is a conceptually distinct feature of social relationships that has received less attention as a pathway to depression [17, 18]. Friends and confidants also capture different aspects of this network. Friends reflect the broader reach of regular contacts outside the family, whereas confidants reflect the narrower core of relationships available for intimate disclosure [19, 20].

Although prior research links unmarried status to depression and points to social support as one explanation, how much of this association is explained by the size of a woman’s social network remains poorly quantified, particularly among women in contemporary Korea. The present study addresses this gap by examining the relationship between marital status and depression in a large national multicenter sample of Korean women. We test the hypothesis that unmarried women are more likely than married women to meet criteria for MDD and that this association is mediated by social network size, defined as the number of friends and confidants. By quantifying the direct and indirect effects of marital status on depression, our goal is to improve understanding of how social relationships relate to women’s mental health in a contemporary Korean society.

## 2. Methods

### 2.1. Participants and Study Design

The Korean Mood Disorder Genetic Study‐Depression (KOMOGEN‐D) project recruited 6049 Korean women (3000 cases with recurrent MDD and 3049 controls with no lifetime major depressive episodes) from 18 hospitals between October 2021 and November 2023 [21]. KOMOGEN‐D is a multicenter genetic study of MDD whose intended target population is Korean women with recurrent depression, together with screened controls without a lifetime depressive episode. In the KOMOGEN‐D study, cases were eligible from age 30 years, whereas controls were recruited only from age 40 years to reduce the likelihood of later incident MDD among participants classified as controls. Therefore, the present analysis was restricted to women aged 40 years or older to align the eligible age range between cases and controls. This yielded 5444 women, and excluding one with a missing marital status left 5443 for analysis. Computer‐assisted interviews were conducted by trained mental health professionals, recorded with consent, and subject to quality control with regular rater training. All procedures received ethical approval from the University of California and local hospital boards.

### 2.2. Measures

Lifetime MDD was assessed with a structured clinical interview administered through a computerized system using items adapted from the Composite International Diagnostic Interview (CIDI 3.0) and expanded to capture the DSM‐5 Criterion A symptoms for a major depressive episode [21]. Marital status was classified into married and unmarried, with the latter including never‐married, divorced/separated, and widowed women; separated was combined with divorced due to small numbers. Education was modeled as a three‐level categorical variable (low for middle school or less, middle for high school, and high for college or higher, including junior college). Heavy episodic drinking was assessed using a single item, asking whether the participant had ever consumed a large quantity of alcohol on a single day, such as two bottles of beer or two‐thirds of a bottle of soju.

Social network size was assessed using two self‐reported count indicators, the number of friends and the number of confidants. Friends were defined by regular contact and some emotional connection, explicitly excluding the spouse, partner, and relatives, and were counted by asking, “How many friends like that do you have?” Confidants were defined as anyone “with whom you have a close relationship and can share your most private feelings” (a friend, a relative, or the spouse) and counted by asking, “How many people have that kind of relationship with you?” Because the two items were asked separately and were not mutually exclusive, overlap between friends and confidants was possible. The raw counts were markedly right‐skewed, with medians of 4 for friends and 2 for confidants but maximum values of 50 and 30, respectively. To reduce the undue influence of extreme upper‐tail values, values above the mean plus three standard deviations were capped, using thresholds calculated in the full cohort before applying the caps. The resulting thresholds were 15.46 for friends and 9.27 for confidants, which were rounded up to integer caps of 16 and 10, respectively. This procedure affected 75 women (1.4%) for friends and 15 women (0.3%) for confidants.

### 2.3. Statistical Analysis

Group differences in continuous variables were tested with independent‐samples *t*‐tests and categorical variables with *χ* ^2^ tests. Multivariable logistic regression estimated (1) the association of marital status with MDD and (2) the association of network size with MDD. All logistic regressions were adjusted for age, education, employment, and heavy episodic drinking, and we report odds ratios (ORs) with 95% confidence intervals (CIs). Because the exposure of interest was the absence of a spouse, the mediation and segmented analyses were based on the dichotomous married‐versus‐unmarried contrast, which also helped preserve statistical power within the strata. The three unmarried subgroups (never married, divorced/separated, and widowed) were examined only descriptively and in the marital‐status regression analyses.

To examine how social networks accounted for the unmarried–married difference in depression, we conducted regression‐based parallel mediation analyses with the marital group as the exposure, number of friends and number of confidants as mediators, and lifetime MDD as the outcome. Linear regression models were fitted for each mediator and a logistic regression model for MDD, including marital group, both mediators, and the same covariates; indirect, direct, and total effects were estimated on the log‐odds scale with 5000 bootstrap resamples to obtain percentile 95% CI.

As an exploratory analysis, we examined possible thresholds in the associations between social network size and MDD using two‐piecewise logistic regression. For friends and confidants separately, we fitted models allowing a single change in slope in the relationship with MDD, adjusting for the same covariates. From these models, we obtained ORs per additional person below and above the estimated breakpoint and formally tested the departures from linearity. We repeated the two‐piecewise (segmented) logistic analyses within the strata of married and unmarried women using the same covariates. All tests were two‐sided with *α* = 0.05, and all analyses were conducted in *R*.

## 3. Results

### 3.1. Baseline Characteristics

In the two‐group comparison (*N* = 5443; married *n* = 3928 and unmarried *n* = 1515), the prevalence of lifetime MDD was nearly twice as high in unmarried women (69.0%) compared with married women (34.3%). Married women also reported larger social networks, with an average of 4.58 friends and 2.61 confidants, compared with 3.51 friends and 1.91 confidants among unmarried women (Table 1).

**Table 1:** Baseline characteristics by marital status (married and unmarried).

Variables	Married (*n* = 3928)	Unmarried (*n* = 1515)	Test	*p*
MDD (%)	1348 (34.3%)	1046 (69.0%)	*χ* ^2^ = 533.69	<0.001
Education (%)	—	—	*χ* ^2^ = 206.97	<0.001
Low	451 (11.5%)	390 (25.7%)	—	—
Mid	1361 (34.7%)	561 (37.0%)	—	—
High	2115 (53.9%)	564 (37.2%)	—	—
Employment (working) (%)	1858 (47.3%)	669 (44.2%)	*χ* ^2^ = 4.22	0.040
Heavy episodic drinking (lifetime) (%)	333 (8.5%)	198 (13.1%)	*χ* ^2 = 25.66^	<0.001
Social network size				
Friends (*n*), *M* ± SD	4.58 ± 3.04	3.51 ± 2.97	*t* = 11.84	<0.001
Confidants (*n*), *M* ± SD	2.61 ± 1.71	1.91 ± 1.76	*t* = 13.19	<0.001
Age *M* ± SD	51.89 ± 7.97	54.62 ± 9.00	*t* = −10.33	<0.001

When marital status was categorized into four groups (married *n* = 3928, never married *n* = 328, divorced/separated *n* = 863, and widowed *n* = 324), married women had the lowest prevalence of MDD (34.3%). Rates were higher among never‐married women (54.6%) and were particularly elevated in divorced or separated (73.9%) and widowed women (70.7%). Mean friend counts were highest in married women (4.58), followed by widowed (4.00), never married (3.54), and divorced or separated (3.32). Mean confidants showed a similar order, with married women reporting 2.61, widowed 2.03, never married 1.96, and divorced or separated 1.85. Other characteristics, such as education, employment, heavy episodic drinking, and age, also varied across groups (Table S1).

### 3.2. Association of Marital Status and Social Network Size With MDD

To examine the relationship between marital status and MDD, we compared each group with married women as the reference. Overall, unmarried women had nearly four‐fold higher odds of MDD (OR 3.99, 95% CI 3.46–4.60). Among the specific categories, divorced or separated women showed the highest odds (OR 4.90, 95% CI 4.09–5.89), followed by never‐married women (OR 3.86, 95% CI 3.00–4.96). Widowed women had the lowest risk among the unmarried groups, though still significantly elevated compared with married women (OR 2.31, 95% CI 1.75–3.07). All differences were statistically significant (Table 2).

**Table 2:** Logistic regression of marital status on MDD.

Group	MDD cases *n* (%)	OR (95% CI)	*p*
Married	1348 (34.3)	1.00 (Ref)	–
Unmarried	1046 (69.0)	3.99 (3.46–4.60)	<0.001
Never married	179 (54.6)	3.86 (3.00–4.96)	<0.001
Divorced/separated	638 (73.9)	4.90 (4.09–5.89)	<0.001
Widowed	229 (70.7)	2.31 (1.75–3.07)	<0.001

*Note*: ORs are relative to married women and adjusted for age, education, employment, and heavy episodic drinking. The percentage in the “MDD cases, *n* (%)” column denotes the proportion of women within each marital‐status group who met criteria for lifetime MDD.

Abbreviation: MDD, major depressive disorder.

We examined whether women with larger social networks had lower odds of MDD. After adjusting for age, education, employment, and heavy episodic drinking, each additional friend was linked to a 13% reduction in odds (OR 0.87, 95% CI 0.85–0.89), and each additional confidant to a 22% reduction (OR 0.78, 95% CI 0.76–0.82). Because the numbers of friends and confidants were modestly correlated (Pearson *r* = 0.37; Spearman *r* = 0.38), we additionally fit a mutually adjusted model (Table 2) including both indicators. Associations attenuated but remained robust, with ORs of 0.90 (95% CI 0.88–0.92) per additional friend and 0.84 (95% CI 0.80–0.87) per additional confidant (Table 3).

**Table 3:** Logistic regression of social network size on MDD.

Variable	Model 1		Model 2	
	Depression OR (95% CI)	*p*	Depression OR (95% CI)	*p*
Friends	0.87 (0.85–0.89)	<0.001	0.90 (0.88–0.92)	<0.001
Confidants	0.78 (0.76–0.82)	<0.001	0.84 (0.80–0.87)	<0.001

*Note*: Association between social network size and major depressive disorder (MDD). Model 1 includes each variable separately (single‐variable models), whereas Model 2 includes number of friends and number of confidants simultaneously in a logistic regression (mutually adjusted). All models adjust for age, education, employment, and heavy episodic drinking.

### 3.3. Mediation Analysis of Marital Status With MDD

To assess the extent to which social network size accounted for the association between marital status and MDD risk, we conducted a parallel mediation analysis adjusted for age, education, employment, and heavy episodic drinking. When the number of friends and confidants was included as mediators, the association between being unmarried and MDD attenuated but remained strong (direct OR ≈ 3.5), while both indirect paths via friends and via confidants were statistically significant. Overall, social network size accounted for only a modest proportion of the unmarried–married difference in MDD risk (≈13%), consistent with partial rather than complete mediation (Table 4).

**Table 4:** Mediation analysis of social network size in the association between marital status and MDD.

Effect	Standardized estimate *β* (95% CI)	S.E.	*Z*‐value	*p*
Direct effect				
Unmarried → MDD	1.25 (1.11–1.40)	0.07	16.8	<0.001
Indirect effect				
Unmarried → friends → MDD	0.10 (0.07–0.13)	0.02	5.85	<0.001
Unmarried → confidants → MDD	0.09 (0.06–0.12)	0.02	5.58	<0.001
Total indirect effect	0.19 (0.14–0.23)	0.02	8.56	<0.001
Component				
Unmarried → friends	−1.09 (−1.27–0.91)	0.09	−11.74	<0.001
Friends → MDD	−0.09 (−0.11–0.06)	0.01	−7.36	<0.001
Unmarried → confidants	−0.61 (−0.71–0.50)	0.05	−11.50	<0.001
Confidants → MDD	−0.15 (−0.19–0.11)	0.02	−7.02	<0.001
Total effect				
Unmarried → MDD	1.43 (1.29–1.58)	0.08	19.10	<0.001

### 3.4. Two‐Piecewise Logistic Regression of Social Network Size

**Figure 1:**
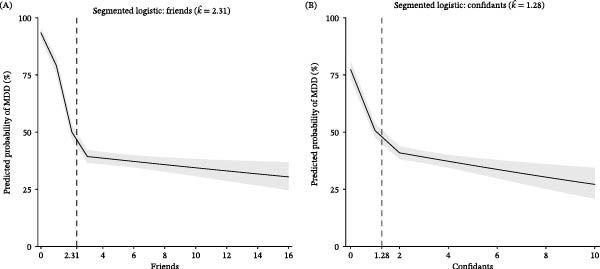
Segmented logistic curves for predicted probability of MDD by (A) number of friends and (B) number of confidants, adjusted for age, education, employment, and heavy episodic drinking. Dashed vertical lines indicate estimated breakpoints (*k̂*) reported in Table 5.

As an exploratory analysis, we used two‐piecewise logistic regression to examine possible thresholds in the association between social network size and MDD. In the overall sample, most of the protective association was concentrated at low network sizes: risk declined steeply up to about 2.31 friends and 1.28 confidants, with little additional benefit beyond these levels (Table 5, Figure 1). When analyses were stratified by marital status, similar thresholds were observed among married women (2.27 friends and 1.17 confidants), whereas among unmarried women, the breakpoints shifted upward (3.51 friends and 3.64 confidants) (Table 5).

**Table 5:** Two‐piecewise logistic regression of social network size and depression.

Variable	Breakpoint (95% CI)	Prebreak OR (95% CI)	Postbreak OR (95% CI)	Davies *p*
Overall				
Friends	2.31 (2.14–2.48)	0.26 (0.21–0.33)	0.97 (0.95–1.00)	<0.001
Confidants	1.28 (1.08–1.47)	0.30 (0.23–0.38)	0.92 (0.88–0.97)	<0.001
Married				
Friends	2.27 (2.06–2.48)	0.29 (0.22–0.38)	0.97 (0.94–1.00)	<0.001
Confidants	1.17 (0.98–1.36)	0.26 (0.19–0.36)	0.94 (0.88–1.00)	<0.001
Unmarried				
Friends	3.51 (2.99–4.03)	0.48 (0.40–0.59)	1.05 (0.98–1.12)	<0.001
Confidants	3.64 (2.63–4.66)	0.67 (0.59–0.76)	1.13 (0.93–1.38)	<0.001

*Note*: Adjusted for age, education, employment, heavy episodic drinking.

## 4. Discussion

Our study found that marital status is significantly correlated with depression in Korean women. Being unmarried, whether never married or no longer married, was associated with roughly four times higher odds of MDD compared with those of being married. Among the unmarried groups, women who were divorced or separated showed the highest odds, nearly five times that of married women, while never‐married and widowed women also had significantly elevated risks. This finding is consistent with prior evidence indicating that married individuals tend to have better mental health outcomes than those who are never married, divorced, or widowed [8]. In our sample, unmarried women not only had greater depression prevalence but also reported markedly smaller social networks, with fewer close friends and emotional confidants compared with those of married women. Importantly, these social network deficits appeared to partially mediate the relationship between marital status and depression.

The partial mediation by social network size highlights the role of social integration in women’s mental health. Unmarried women in this study had significantly fewer friends and confidants, and those with smaller social networks showed a higher likelihood of depression. This aligns with prior research showing that women with low social network size and intimacy have elevated depressive symptoms [22]. A low quantity or quality of social relationships can lead to loneliness and social isolation, which are known risk factors for depression [23]. On the other hand, social support is a well‐established protective factor against depression, with the strongest effects seen in vulnerable populations [24]. In this context, one reason unmarried women have a greater risk of depression is their reduced access to supportive social relationships.

One likely explanation for the smaller social networks among unmarried women is that relationship formation and maintenance differ across adulthood. Marriage combines two individuals’ networks and generates frequent contact through recurrent family routines and rituals, which sustain ties that are both numerous and close [25, 26]. Marriage provides opportunities for social participation through spouses, in‐laws, and family‐centered activities, which help sustain larger and closer networks [27]. Moreover, one partner’s social engagement often reinforces the other’s satisfaction and participation [28]. Because these encounters are embedded in everyday life, married women may need comparatively less time and effort to keep their relationships active.

In contrast, unmarried women have fewer ways to sustain networks, particularly in cultures where the family is the main setting for social life [4]. To achieve comparable levels of contact, they need to make conscious plans, and in family‐centered settings, they have far fewer natural opportunities [29]. As peers marry, priorities and conversation topics may shift toward family and children, and social gatherings tend to be reorganized around couples and child‐related activities. In this process, unmarried women are often gradually pushed aside, leading to less frequent and less close contact. As the proportion of married individuals grows, these exclusionary dynamics intensify, amplifying social isolation and the risk of depression [9]. Our findings accord with this interpretation, suggesting that part of the mental health advantage associated with marriage stems from the social structure it provides.

One interesting point of this study is the presence of network thresholds. The segmented models indicate a practical minimum of two friends and one confidant for a substantial reduction in depression risk, suggesting a sufficiency level of social connection. This nonlinear effect underscores that it may not be the total number of social contacts that matters. Rather, what matters is ensuring at least a minimum core of supportive relationships [30]. Notably, these thresholds differed by marital status. While married women needed roughly two friends and one close confidant, unmarried women required three to four friends or confidants to achieve a comparable reduction in depression risk. As discussed above, an unmarried woman may rely on multiple confidants or a broader set of friends to compensate for the support that a partner would otherwise provide [31].

It is also important to acknowledge that the relationship between marital status and depression may not be purely environmental. Recent behavioral genetic research indicates that divorce is heritable and shares genetic liabilities with major depression. For instance, large‐scale adoption data suggest that genetic factors contribute to the transmission of divorce [32], while genetic profile studies show that divorced individuals carry elevated risks for internalizing disorders [33]. This suggests that our findings may partially reflect gene–environment correlations, where genetic traits influence both marital instability and depression. However, regardless of genetic confounding, the mediating role of social network size remains clinically significant. While genetic risks are not modifiable, social isolation acts as a proximal pathway that can be targeted for intervention.

This study has several limitations. First, the cross‐sectional design precludes definitive causal inference. We cannot determine temporal directionality or rule out reverse causality, where depression might lead to marital dissolution or social withdrawal [13]. Second, the unmarried category comprises heterogeneous subgroups of never married, divorced, and widowed. While this allowed us to examine the broad impact of spousal absence, we acknowledge that psychosocial mechanisms differ across never‐married, divorced, and widowed women. Third, network size was assessed by self‐report, which may be subject to recall or reporting bias, particularly in depressed individuals. Fourth, as the sample was restricted to Korean women, the findings may not generalize to men or other cultural contexts. In addition, because the analytic sample was restricted to women aged 40 years or older, the findings may not generalize to younger women. The roles of marital status, friends, and confidants in relation to depression may vary across the life course as social relationships earlier in adulthood may be less family‐centered than those in later life. Therefore, our estimates should be interpreted within this older age range. Finally, we could not fully control for all potential confounders. Residual confounding from unmeasured psychosocial factors or shared genetic liabilities between marital instability and depression [32, 33] may partly account for the observed associations.

Despite these limitations, this study highlights important implications. Part of the association between marital status and depression appears to operate through the size of one’s network. Thus, assessing both marital status and the size and strength of social networks provides an important enhancement to traditional measures of depression risk. We establish a protective baseline of social connection at approximately two friends and one confidant, providing a clinically interpretable threshold for this risk. By mapping both the pathways and the sufficiency levels of connection, this study offers a clear framework for understanding why social integration matters for women’s mental health and how differences by marital status arise.

## Author Contributions

Han‐Sung Lee conceptualized the study, developed the methodology, analyzed the data, and wrote the original manuscript. Yong Min Ahn, Sang Jin Rhee, and Jonathan Flint acquired the funding and supervised the research. Kenneth Kendler, Sang Jin Rhee, and Jonathan Flint also contributed to the study conceptualization. Sooyeon Min and Yoojin Song curated the data and administered the project. Heon‐Jeong Lee, Seunghee Won, Kyu Young Lee, Dongyun Lee, Do Hoon Kim, Ji Hyun Baek, Young‐Hoon Ko, Kwang‐Yeon Choi, Young Tak Jo, Sung Joon Cho, Hong Seok Oh, Won Sub Kang, Young Min Choe, Sungwon Roh, Hyun‐Ju Kim, Joonho Choi, Ga Eun Kim, So‐Hyun Ahn, Hyeon‐Ah Lee, and Ji‐Woon Jeong provided resources and conducted the investigation. Kenneth Kendler, Sang Jin Rhee, Ji‐Woon Jeong, and Jonathan Flint validated the findings, reviewed, and edited the manuscript.

## Funding

This work was supported by the National Institutes of Health (Grant U01MH126798) and the Wellcome Trust (Grant 200176/A/15/Z).

## Disclosure

All authors reviewed and approved the final submission. The funding sources had no role in the study design, collection, analysis and interpretation of data, writing of the report, or in the decision to submit the article for publication.

## Ethics Statement

The study protocol was approved centrally by the Institutional Review Board at the University of California, Los Angeles, and by the ethics committees of all participating hospitals in South Korea, including Seoul National University Hospital (IRB Number 2101‐193‐1194).

## Conflicts of Interest

The authors declare no conflicts of interest.

## Supporting Information

Additional supporting information can be found online in the Supporting Information section.

## Supporting information


**Supporting Information** Table S1 presents the baseline characteristics of participants by detailed marital status (married, never married, divorced/separated, and widowed), expanding the dichotomous classification (married vs. unmarried) used in the main analysis.

## Data Availability

The data that support the findings of this study are available upon request from the corresponding author. The data are not publicly available due to privacy or ethical restrictions.

## References

[1] Herre B. , Samborska V. , Ortiz-Ospina E. , and Roser M. , Marriages and Divorces. Our World in Data, 2020, (accessed February 2026) https://ourworldindata.org/marriages-and-divorces.

[2] Hwang J. , Later, Fewer, None? Recent Trends in Cohort Fertility in South Korea, Demography. (2023) 60, no. 2, 563–582, 10.1215/00703370-10585316. 36847264

[3] Raymo J. M. and Park H. , Marriage Decline in Korea: Changing Composition of the Domestic Marriage Market and Growth in International Marriage, Demography. (2020) 57, no. 1, 171–194, 10.1007/s13524-019-00844-9. 31919807 PMC7382948

[4] Raymo J. M. , Park H. , Xie Y. , and Yeung W.-J. J. , Marriage and Family in East Asia: Continuity and Change, Annual Review of Sociology. (2015) 41, no. 1, 471–492, 10.1146/annurev-soc-073014-112428. PMC6070151 30078932

[5] Yoo S. H. and Sobotka T. , Ultra-Low Fertility in South Korea: The Role of the Tempo Effect, Demographic Research. (2018) 38, 549–576, 10.4054/DemRes.2018.38.22.

[6] Kim J. and Kim T. , Family Formation and Dissolution During the COVID-19 Pandemic: Evidence From South Korea, Global Economic Review. (2021) 50, no. 1, 1–19, 10.1080/1226508X.2021.1874466.

[7] Yan X. Y. , Huang S. M. , Huang C. Q. , Wu W. H. , and Qin Y. , Marital Status and Risk for Late Life Depression: A Meta-Analysis of the Published Literature, Journal of International Medical Research. (2011) 39, no. 4, 1142–1154, 10.1177/147323001103900402. 21986116

[8] Zhai X. , Tong H. H. Y. , and Lam C. K. , et al.Association and Causal Mediation Between Marital Status and Depression in Seven Countries, Nature Human Behaviour. (2024) 8, no. 12, 2392–2405, 10.1038/s41562-024-02033-0. 39496771

[9] Lee J. , Kim H. , and Woo J. , et al.Impacts of Remaining Single Above the Mean Marriage Age on Mental Disorders and Suicidality: A Nationwide Study in Korea, Journal of Korean Medical Science. (2020) 35, no. 37, 10.3346/jkms.2020.35.e319. PMC7505730 32959544

[10] Cherlin A. J. , The Deinstitutionalization of American Marriage, Journal of Marriage and Family. (2004) 66, no. 4, 848–861, 10.1111/j.0022-2445.2004.00058.x.

[11] Liu H. and Umberson D. J. , The Times They Are a Changin’: Marital Status and Health Differentials From 1972 to 2003, Journal of Health and Social Behavior. (2008) 49, no. 3, 239–253, 10.1177/002214650804900301. 18771061 PMC3150568

[12] Vaingankar J. A. , Abdin E. , and Chong S. A. , et al.The Association of Mental Disorders With Perceived Social Support, and the Role of Marital Status: Results From a National Cross-Sectional Survey, Archives of Public Health. (2020) 78, no. 1, 10.1186/s13690-020-00476-1, 108. 33133595 PMC7592592

[13] Teo A. R. , Choi H. , and Valenstein M. , Social Relationships and Depression: Ten-Year Follow-up From a Nationally Representative Study, PLoS ONE. (2013) 8, no. 4, 10.1371/journal.pone.0062396. PMC3640036 23646128

[14] Caetano S. C. , Silva C. M. , and Vettore M. V. , Gender Differences in the Association of Perceived Social Support and Social Network With Self-Rated Health Status Among Older Adults: A Population-Based Study in Brazil, BMC Geriatrics. (2013) 13, no. 1, 10.1186/1471-2318-13-122, 122. 24229389 PMC4225700

[15] Shin H. and Park C. , Gender Differences in Social Networks and Physical and Mental Health: Are Social Relationships More Health Protective in Women Than in Men?, Frontiers in Psychology. (2023) 14, 10.3389/fpsyg.2023.1216032, 1216032. 38213610 PMC10782512

[16] Kendler K. S. , Myers J. , and Prescott C. A. , Sex Differences in the Relationship Between Social Support and Risk for Major Depression: A Longitudinal Study of Opposite-Sex Twin Pairs, American Journal of Psychiatry. (2005) 162, no. 2, 250–256, 10.1176/appi.ajp.162.2.250. 15677587

[17] House J. S. , Landis K. R. , and Umberson D. , Social Relationships and Health, Science. (1988) 241, no. 4865, 540–545, 10.1126/science.3399889. 3399889

[18] Berkman L. F. , Glass T. , Brissette I. , and Seeman T. E. , From Social Integration to Health: Durkheim in the New Millennium, Social Science & Medicine. (2000) 51, no. 6, 843–857, 10.1016/S0277-9536(00)00065-4. 10972429

[19] Granovetter M. S. , The Strength of Weak Ties, American Journal of Sociology. (1973) 78, no. 6, 1360–1380, 10.1086/225469.

[20] Marsden P. V. , Core Discussion Networks of Americans, American Sociological Review. (1987) 52, no. 1, 122–131, 10.2307/2095397.

[21] Min S. , Rhee S. J. , and Song Y. , et al.Deep Phenotyping at Scale: Study Protocol for the Korean Mood Disorder Genetic Study-Depression (KOMOGEN-D), American Journal of Medical Genetics Part B: Neuropsychiatric Genetics. (2026) 201, no. 2, 105–115, 10.1002/ajmg.b.33056. 40851433

[22] Goldberg E. L. , Van Natta P. , and Comstock G. W. , Depressive Symptoms, Social Networks and Social Support of Elderly Women, American Journal of Epidemiology. (1985) 121, no. 3, 448–456, 10.1093/oxfordjournals.aje.a114017. 3874542

[23] Ge L. , Yap C. W. , Ong R. , and Heng B. H. , Social Isolation, Loneliness and Their Relationships With Depressive Symptoms: A Population-Based Study, PLoS ONE. (2017) 12, no. 8, 10.1371/journal.pone.0182145. PMC5568112 28832594

[24] De Risio L. , Pettorruso M. , and Collevecchio R. , et al.Staying Connected: An Umbrella Review of Meta-Analyses on the Push-and-Pull of Social Connection in Depression, Journal of Affective Disorders. (2024) 345, 358–368, 10.1016/j.jad.2023.10.112. 37852587

[25] Haggerty B. B. , Du H. , Kennedy D. P. , Bradbury T. N. , and Karney B. R. , Stability and Change in Newlyweds’ Social Networks Over the First Years of Marriage, Journal of Family Psychology. (2023) 37, no. 1, 20–30, 10.1037/fam0001016. 35862079 PMC9942941

[26] Kalmijn M. , Shared Friendship Networks and the Life Course: An Analysis of Survey Data on Married and Cohabiting Couples, Social Networks. (2003) 25, no. 3, 231–249, 10.1016/S0378-8733(03)00010-8.

[27] Piechota A. , Ali T. , Tomlinson J. M. , and Monin J. K. , Social Participation and Marital Satisfaction in Mid to Late Life Marriage, Journal of Social and Personal Relationships. (2022) 39, no. 4, 1175–1188, 10.1177/02654075211056289. 35529021 PMC9074824

[28] Ang S. , Your Friends, My Friends, and Our Family: Informal Social Participation and Mental Health Through the Lens of Linked Lives, Social Science & Medicine. (2021) 276, 10.1016/j.socscimed.2021.113848, 113848. 33770570

[29] Girme Y. U. , Park Y. , and MacDonald G. , Coping or Thriving? Reviewing Intrapersonal, Interpersonal, and Societal Factors Associated With Well-Being in Singlehood From a Within-Group Perspective, Perspectives on Psychological Science. (2023) 18, no. 5, 1097–1120, 10.1177/17456916221136119. 36534959 PMC10475216

[30] Thompson A. , Smith M. A. , McNeill A. , and Pollet T. V. , Friendships, Loneliness and Psychological Wellbeing in Older Adults: A Limit to the Benefit of the Number of Friends, Ageing and Society. (2024) 44, no. 5, 1090–1115, 10.1017/S0144686X22000666.

[31] Adamczyk K. , An Investigation of Loneliness and Perceived Social Support Among Single and Partnered Young Adults, Current Psychology. (2016) 35, no. 4, 674–689, 10.1007/s12144-015-9337-7. 27891044 PMC5104760

[32] Salvatore J. E. , Larsson Lönn S. , Sundquist J. , Sundquist K. , and Kendler K. S. , Genetics, the Rearing Environment, and the Intergenerational Transmission of Divorce: A Swedish National Adoption Study, Psychological Science. (2018) 29, no. 3, 370–378, 10.1177/0956797617734864. 29346036 PMC5854499

[33] Salvatore J. E. , Ohlsson H. , Sundquist J. , Sundquist K. , and Kendler K. S. , Family Genetic-Risk Profiles Associated With Divorce, Clinical Psychological Science. (2024) 12, no. 6, 1162–1178, 10.1177/21677026231214204. 39582791 PMC11583951

