# Supplementary material for: Friend and Confidant Thresholds: Social Network Size as a Mediator Between Marital Status and Major Depressive Disorder
Source: Depress Anxiety. 2026 Jul 5;2026:5174978. doi: 10.1155/da/5174978 (PMC13333932; doi:10.1155/da/5174978)
Supplement: Supplementary file 1 — Supporting Information Table S1 presents the baseline characteristics of participants by detailed marital status (married, never married, divorced/separated, and widowed), expanding the dichotomous classification (married vs. unmarried) used in the main analysis. [file DA-2026-5174978-s001.docx]

**Supplementary Table S1. Baseline characteristics by marital status (Married, Never married, Divorced/Separated, Widowed)**

| Variables | Married (n=3928) | Never married (n=328) | Divorced/Separated (n=863) | Widowed (n=324) | Test | p |
| --- | --- | --- | --- | --- | --- | --- |
| MDD, % | 1348 (34.3%) | 179 (54.6%) | 638 (73.9%) | 229 (70.7%) | χ²=571.68 | < 0.001 |
| Education, % |  |  |  |  | χ²=475.44 | < 0.001 |
| low | 451 (11.5%) | 21 (6.4%) | 220 (25.5%) | 149 (46.0%) |  |  |
| mid | 1361 (34.7%) | 86 (26.2%) | 369 (42.8%) | 106 (32.7%) |  |  |
| high | 2115 (53.9%) | 221 (67.4%) | 274 (31.7%) | 69 (21.3%) |  |  |
| Employment (working), % | 1858 (47.3%) | 197 (60.1%) | 352 (40.8%) | 120 (37.0%) | χ²=48.24 | < 0.001 |
| Heavy episodic drinking (lifetime), % | 333 (8.5%) | 52 (15.9%) | 122 (14.1%) | 24 (7.4%) | χ²=41.99 | < 0.001 |
| Social network size |  |  |  |  |  |  |
| Friends (n), M ± SD | 4.58 ± 3.04 | 3.54 ± 2.71 | 3.32 ± 2.81 | 4.00 ± 3.52 | F=55.10 | < 0.001 |
| Confidants (n), M ± SD | 2.61 ± 1.71 | 1.96 ± 1.63 | 1.85 ± 1.75 | 2.03 ± 1.90 | F=59.71 | < 0.001 |
| Age, M ± SD | 51.89 ± 7.97 | 47.89 ± 7.23 | 54.56 ± 7.94 | 61.58 ± 8.02 | F=209.50 | < 0.001 |
